# Neuroanatomic Correlates of Distance and Direction Processing During Cognitive Map Retrieval

**DOI:** 10.3389/fnbeh.2020.00130

**Published:** 2020-08-24

**Authors:** Igor Faulmann, Virginie Descloux, Arnaud Saj, Roland Maurer

**Affiliations:** ^1^Frontiers Media SA, Lausanne, Switzerland; ^2^Faculty of Psychology and Educational Sciences, University of Geneva, Geneva, Switzerland; ^3^Ecole Doctorale en Neurosciences Lémaniques, Université de Lausanne, Geneva, Switzerland; ^4^Fribourg Cantonal Hospital, Fribourg, Switzerland; ^5^Département de Psychologie, Faculté des Arts et des Sciences, Université de Montréal, Montreal, QC, Canada; ^6^CRIR/Institut Nazareth et Louis-Braille du CISSS de la Montérégie-Centre, Longueuil, QC, Canada

**Keywords:** cognitive map, evaluation of distance, evaluation of direction, cognitive map reading test, fMRI, hippocampus

## Abstract

Navigating toward a goal and mentally comparing distances and directions to landmarks are processes requiring reading information off the memorized representation of the environment, that is, the cognitive map. Brain structures in the medial temporal lobe, in particular, are known to be involved in the learning, storage, and retrieval of cognitive map information, which is generally assumed to be in allocentric form, whereby pure spatial relations (i.e., distance and direction) connect locations with each other. The authors recorded functional magnetic resonance imaging activity, while participants were submitted to a variant of a neuropsychological test (the Cognitive Map Reading Test; CMRT) originally developed to evaluate the performance of brain-lesioned patients and in which participants have to compare distances and directions in their mental map of their hometown. Our main results indicated posterior parahippocampal, but not hippocampal, activity, consistent with a task involving spatial memory of places learned a long time ago; left parietal and left frontal activity, consistent with the distributed processing of navigational representations; and, unexpectedly, cerebellar activity, possibly related to the role of the cerebellum in the processing of (here, imaginary) self-motion cues. In addition, direction, but not distance, comparisons elicited significant activation in the posterior parahippocampal gyrus.

## Introduction

Spatial cognition consists in a plethora of high-level cognitive abilities; among them, the ability to learn and to navigate in large-scale environments is probably one of the most complex skills. Navigation is here defined as a moving behavior specifically oriented toward a known location, as opposed to the exploration of unknown areas of one’s environment.

In the framework defined by O’Keefe and Nadel (1978), the so-called *cognitive map* is thought to be an *allocentric*—that is, viewpoint independent—representation of our environment, built progressively during exploration. The hippocampal *place cells* (O’Keefe and Nadel, 1978; Ekstrom et al., 2003) are potentially the actual neural substrate of the *cognitive map*, together with neurons in neighboring structures, in particular grid cells in the entorhinal cortex (for a review, see Barry and Burgess, 2014).

Many recent studies point to a predominant hippocampal and parahippocampal role in spatial cognition, as well as in the more specific cluster of navigational skills, be it for exploration or navigation *per se* (e.g., Wolbers and Büchel, 2005; for a review, see Burgess, 2008). More recently, hippocampal size has even been shown to predict at which pace a *cognitive map* was learned (Schinazi et al., 2013). Similarly, Nedelska et al. (2012) found in humans a significant correlation between hippocampal size and spatial navigation performance in humans, in both a real-space and a virtual Morris water maze (although this correlation was significant only in amnesic patients diagnosed with mild cognitive impairment and mild and moderate Alzheimer disease, and not on cognitively intact older controls).

Furthermore, a double dissociation between the anterior and the posterior hippocampus has been observed (Iaria et al., 2007; Schinazi et al., 2013): activity in the former is specifically related to the *learning phase*, whereas activity in the latter is more related to the *reading phase*, that is, the moment when information is recalled from the cognitive map.

Epstein (2008) concluded in his review article that the parahippocampal cortex plays a mandatory role in active navigation (e.g., Rosenbaum et al., 2004; Rauchs et al., 2008). In the same line, the results obtained by Park et al. (2007), and more recently by Bastin et al. (2013), also show a parahippocampal role in navigational skills. This may be actually linked to the second cell type thought to play a great role in navigation: the *grid cells*. In the human brain, they seem to be located—among other regions—in the parahippocampal cortex, entorhinal cortex, and in the subicular region (Hafting et al., 2005; Doeller et al., 2010).

Moreover, the hippocampus and the parahippocampal structures (i.e., parahippocampal cortex, subicular, and entorhinal regions), as well as the retrosplenial (Burgess, 2008; Epstein, 2008) and prefrontal cortices (Silk et al., 2010), seem necessary for spatial cognition. To what extent it is so for the storage, recovery, and usage of these representations remains more debated (Ekstrom et al., 2014).

Indeed, some results tend to show a greater implication of these structures for the construction of a cognitive map, compared to a lesser role for the storage, usage, and recovery phases (Rosenbaum et al., 2004; Shrager et al., 2007, 2008). This idea is actually consistent with findings of Schinazi et al. (2013), who found that hippocampal size predicted the pace of the cognitive map learning phase, which again is consistent with a more prominent role in the learning phase than in the usage or recovery phase (although this double dissociation has neither been specifically studied nor observed by the authors).

Recently, Ekstrom et al. (2014) argued in their review that the hippocampus seems not to be the only necessary structure for allocentric processes underlying navigation. They rather proposed in their nonaggregate network model a network-based computation of interacting brain structures, namely, the hippocampus and the parahippocampal, retrosplenial, prefrontal, and parietal cortices.

In another review, Wolbers and Wiener (2014) argue that the strict dissociation, made by many, between, on one side, the parietal/striatal circuits involved in egocentric computing vs., on the other hand, the entorhinal/hippocampal circuits involved in allocentric computing, is actually not compatible with a growing number of experimental data. These authors distinguish between the vista space, which can be globally visualized from a single location or with little exploration only (e.g., single room or a city square), and environmental spaces, such as neighborhoods or towns, which cannot be apprehended from a single point of view, but require extended exploration. They point out the facts that: (1) a determinant role is played by the scale of space (vista space vs. environmental space) used in a given study on the cognitive process recruited, something that, of course, can impact which brain structure is used; and that (2) the variation between studies concerning the (egocentric vs. allocentric) frames involved in the tasks complicates the interpretation of neurophysiological data.

Although it is compatible with the aforementioned theories, Spiers and Maguire (2007) framework does not explicitly make use of concepts like cognitive map or egocentric vs. allocentric representations. They suggested the existence of a large scale (i.e., when the goal location is not visible) navigational guidance system, with goal distance and goal direction coded separately and specifically in the brain. According to these authors, spatial information from hippocampal place cells is needed to guide behavior toward a location, but is not sufficient in and of itself for large-scale navigation. This spatial information is rather integrated with goal-related information, downstream from the hippocampus. Spiers and Maguire (2007) used a virtual simulation of London in a functional magnetic resonance imaging (fMRI) study, where subjects (licensed taxi drivers) had to navigate to goal destinations. They indeed observed a dichotomy between distance and direction, in structures downstream of the hippocampus. They found that medial prefrontal cortex activity correlated positively with goal proximity, whereas subicular/entorhinal activity correlated negatively with it (i.e., the closer the participants get to the goal, the more their medial prefrontal cortex is active, and the less their subicular and entorhinal regions are). For goal direction, a positive correlation was found between bilateral posterior parietal cortex activity and the egocentric direction to the goal (i.e., the smaller the angle between the current egocentric direction and the direction to the goal, the less the posterior parietal cortex is active). Interestingly, they did not find any active voxels at the hippocampal level, which is in accordance with the aforementioned hypothesis (i.e., the hippocampus is not *per se* responsible for navigation).

In an attempt to assess cognitive processes underlying specifically the two primitives of large-scale navigational process (i.e., goal distance and direction), we used a recently designed ecological task called the Cognitive Map Recall Test (CMRT). This task, now validated as part of a three-test set (Descloux et al., 2015; Descloux and Maurer, 2018), was originally developed to assess behavioral differences between healthy subjects and patients with possible topographical disorientation (Aguirre and D’Esposito, 1999), with an emphasis on its ecological properties and its ability to evaluate navigational skills in well-known, large-scale environments. More specifically, this task requires participants to make judgments about distances and directions in their familiar environment (see “*Methods*” section for details).

Descloux (2013) observed no differences in accuracy between judgment about distances and judgment about directions in healthy subjects, while there were significant differences in patients. More specifically, while patients with right posterior lesions were impaired in both categories of questions relative to healthy subjects, they were more impaired in evaluating directions than distances.

By using VLSM [voxel-based lesion-symptom mapping, a technique assessing statistical relationships between specific damaged brain regions and subsequent behavioral deficits (Bates et al., 2003)], Descloux (2013) also inferred the differential neuroanatomic substrates underlying the various response patterns. More precisely, focal lesions in the right anterior parahippocampal gyrus and right insula cause difficulties specifically for the evaluation of distances, whereas larger temporal and parietal lesions cause specific difficulties with directions.

Our main goal with this study is to investigate the neuroanatomic correlates of the processes involved in distance and direction computations while retrieving cognitive map information, during the execution of the CMRT. An ancillary goal is to determine whether this task, which implies a form of mental navigation, taps into the same resources as real navigation. If it indeed does, we expect hippocampal, parahippocampal, and parietal activation, reflecting structures activated during real navigation; we also expect higher activation in the right hemisphere (Javadi et al., 2017).

## Methods

This research has been approved by the Faculty Ethical Committee of the Psychology and Educational Sciences Faculty, Geneva University. All subjects gave their written consent for participation and use of their data, provided they would be rendered anonymous and averaged, for educational and publication purposes.

### Subjects

Twenty-three subjects participated in the experiment (11 women, 12 men); they were recruited among the experimenter’s acquaintances; they were aged between 21 and 61 (mean = 29.04, *SD* = 8.94), and only one was left-handed. Everyone had lived in Geneva for at least 2 years.

### Scanning Protocol and Apparatus

Magnetic resonance imaging data were acquired in the Brain and Behavior Laboratory at University Medical Center, using a 3-T whole-body Siemens MAGNETOM TrioTim syngo MR B17 system with the standard head-coil configuration. A four-button response box, laid on the subject’s chest, was used to record responses.

For each participant, a high-resolution anatomical image was acquired before the functional scans, using a T1-weighted sequence [field of view (FOV) = 256 mm, repetition time (TR)/echo time (TE)/flip angle = 1,900 ms/2.27 ms/9°, slice thickness = 1 mm]. This anatomical image was used for coregistration with functional images and subsequent normalization procedure.

Functional T2*-weighted images were obtained using echoplanar imaging (EPI) with axial slices (*FOV* = 205 mm, TR/TE/flip angle = 2,100 ms/30 ms/80°, slice thickness = 3.2 mm). Each functional volume comprised 36 contiguous slices, parallel to the inferior surface of occipital and temporal lobes, with a final voxel size of 3.2 × 3.2 × 3.2 mm.

All fMRI data were processed and analyzed using the general linear model for event-related designs in SPM8 (Wellcome Department of Imaging Neuroscience, London, UK^1^). Functional images were realigned, corrected for slice timing, normalized to an EPI template, and spatially smoothed (5-mm full-width at half-maximum). Statistical analyses were performed on a voxelwise basis across the whole brain.

Parameter estimates for each regressor were estimated at each voxel by general linear model (GLM) using a least-squares fit to the data, for each condition and each individual participant. Statistical parametric maps of the *t*-statistic [SPM(T)] generated from linear contrasts between conditions in individual subjects were then included in a second-stage random-effects analysis, using one-sample *t-tests* on the contrast images obtained from each condition in each participant. The resulting random-effects maps SPM(T) was thresholded voxelwise at conventional statistical values (*p* < 0.001 uncorrected, with a cluster threshold of *p* < 0.05). Main comparisons were performed between each condition (distance and direction) and 0, between conditions, and for male vs. female participants. Activation results were visualized using the xjView toolbox for MATLAB.

### Task and Stimuli

Prior to the fMRI scanning, subjects were individually contacted in order to determine if each of them knew perfectly where the landmarks we preselected were located (they were chosen from among a set of common places in the city, such as the central train station, the main hospital, the university…). To do so, they were asked the question, “Do you know exactly where the […] is?” for each landmark. If the participant hesitated, or if the answer was anything other than “yes,” we considered that the participant did not really or clearly know where this landmark is.

The CMRT consists in a mental comparison between either distances or angles, which are both determined using those known landmarks. Subjects are first asked to imagine themselves at a *reference point*, which is here a known landmark in the city (e.g., “Imagine yourself in front of the train station”).

Then, for the *distance comparison* condition, the subjects are given the names of two other distant, known landmarks (that could not be seen even if the participant was physically at the reference point and looking in the right direction; further referred to as *targets*). They must choose which of the two targets is farther from the reference point.

For the *direction comparison* condition, an orienting, or *reference*, direction must first be defined. Subjects are therefore given a second distant landmark, and they must imagine being oriented toward it (e.g., participants could be asked to imagine themselves standing in front of the train station, additionally facing the Natural History Museum, which lies 1.7 km away from the station). Subjects are then given the names of two other distant targets, and they are asked for which of those they would have to rotate more in order to face it. For instance, for the reference direction we just gave them (i.e., from the train station facing the Natural History Museum), the instruction could be: “Would you have to rotate more to face the university building or the hospital?” (for further details on this task, see Descloux and Maurer, 2018).

Sets of three, respectively four, locations for the test were generated, by means of *ad hoc* software, with several constraints ensuring that items would be neither too easy nor too hard to solve. More specifically, for the distance condition, the larger distance was between 1.3 and 1.7 times bigger than the smaller one, and the two directions from the reference point to the targets were at least 45° apart, in order to avoid an alignment, which could be simpler to solve and may involve sequential processing rather than cognitive map readout For the direction condition, the difference between the smallest and the largest rotation was between 45° and 60°, and measured from the given reference direction, every required mental rotation was between 45° and 135°.

Based on the set of common landmarks and the aforementioned constraints, we were then able to build four equivalent versions of the task, matched to each participant’s knowledge (i.e., each participant knew every landmark he/she was asked to process during the task).

While the original CMRT (Descloux and Maurer, 2018) was presented orally to the subjects, we adapted it for written presentation on a screen for use in the MRI scanner. We used E-prime/E-run 2.0 to present the questions on a back-projection screen just outside the scanner. The subjects could read them by means of a 45° tilted mirror set right in front of their eyes while they were lying in the scanner.

We further adapted the CMRT to be usable in an fMRI block design. The run had a total of five blocks of 10 items each; each block was split into two miniblocks of five items (one miniblock per condition, i.e., five were direction comparison items and five were distance comparison items; items from the two conditions were not mixed, and items within a miniblock were randomized). Miniblock order was neutralized (the first block contained distance item and then direction items; the second contained direction items and then distance items, etc.), and each item was presented only once to avoid a learning effect.

At the beginning of each miniblock, a slide would first ask the subject to imagine being in the required location (and, for directions, oriented toward a specific landmark) and would show the question, for example, “Imagine you are standing in front of the station, which is the farthest location? Press a key to continue.” This slide was presented only once.

Upon key press by the subject, a fixation cross centered on the screen appeared during 500 ms. This was followed by the alternative, for example, “the church or the parking?” The subjects had to use button 1 for the first choice, and button 2 for the second choice. After their response, the next item was presented, beginning again with the fixation cross. If subjects took more than 15 s to respond, the current item was automatically discarded, and the next one was presented.

On the upper part of each slide, the reference landmark—for direction items, the *two* landmarks defining the reference direction—was always printed as a reminder. This precaution was taken to allow participants to keep in mind the reference(s) point(s) as the block proceeded, because five items followed one another without intervening instruction screen.

## Results

### Behavioral Results

We wanted to check that the two conditions (*distance* and *direction*, henceforth referred to as DIST and DIR, respectively) were equivalent in difficulty. To assess this point, a repeated-measures analysis of variance was used (with condition as independent variable). The difference in the number of correct responses in conditions DIST (mean = 16.61, *SD* = 2.71) and DIR (mean = 16.91, *SD* = 3.41) was not statistically significant (*F*_(1, 22)_ = 0.17, *P* = 0.69). However, the response time was significantly different in conditions DIST (mean = 5, 444.86 ms, *SD* = 1, 416.32) and DIR (mean = 6, 085.93 ms, *SD* = 1, 492.50): (*F*_(1, 22)_ = 14.62, *P* < 0.001). Additionally, correct response rates were statistically different from chance level for both conditions DIST (66.44%) and DIR (67.64%): (*P* < 0.001 for both conditions).

### fMRI Results

Standard realignment, coregistration, normalization, and smoothing (Ashburner et al., 2013) were applied on raw data. Additionally, in order to focus more specifically on brain activation subtending the operations on the cognitive map, we only kept the MRI signals associated with correct responses.

All fMRI results were obtained with an uncorrected *p*-value of 0.001 and a cluster size threshold of 8 (which means that any cluster containing fewer than eight voxels does not appear). Below are given their MNI *x, y*, and *z* coordinates and *Z* scores.

**Figure 1:**
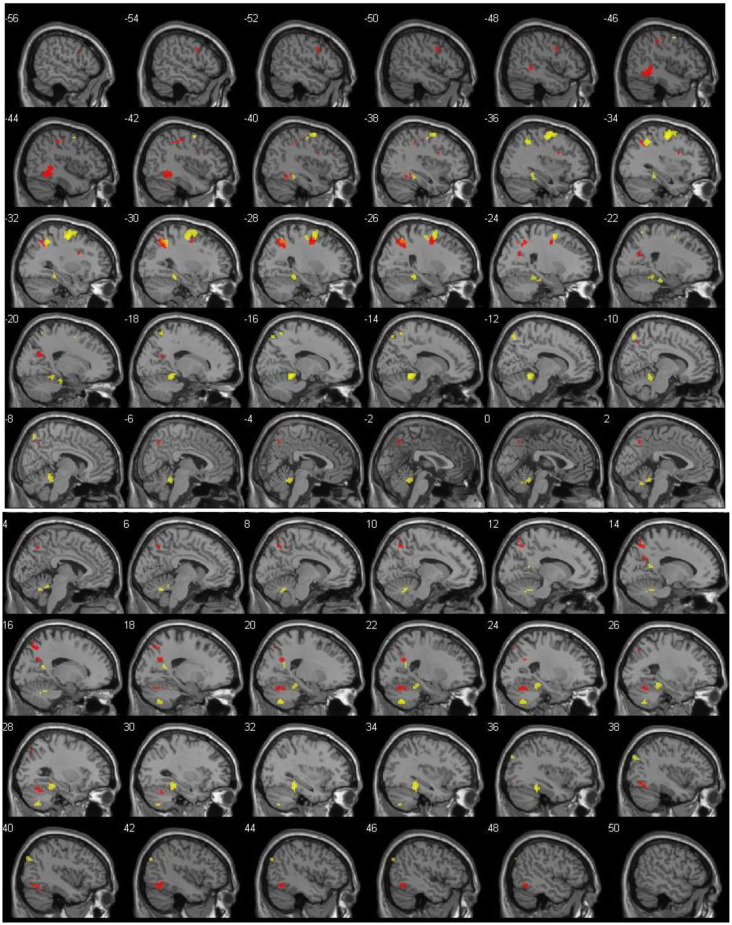
Left (top) and right (bottom) hemisphere activation (*p* = 0.001, uncorrected), shown with 2-mm spacing between each slice. Value on the top left of each slice shows *x* coordinate. Red voxels show suprathreshold activation for distance (DIST) condition, whereas yellow voxels stand for direction (DIR) condition. Top left number of each slice indicate MNI *x* coordinate.

Results for DIST-only and DIR-only activations (compared to 0) can be viewed on Table 1 and Figure 1 and are described below. All other contrasts (DIST–DIR and female–male) showed no significant results.

**Table 1:** Peak MNI coordinates, split between each region, then between distance and direction (DIST and DIR).

		Region		*xyz* Peak coordinates	BA location of peak voxel	Cluster size
Frontal	DIST	iFO	L	−33/8/28	N/A	9
		iFG	L	−51/5/34	9	14
		sFL	L	−24/−4/46	N/A (6)	27
	DIR	PreCG	L	−33/−10/58	6 (4)	133
Temporal	DIST	iTL	R	45/−58/−14	N/A (37)	44
		TL	L	−45/−40/1	N/A (37)	63
	DIR	FG	R	33/−37/−17	N/A (20/36/37)	73
		CC	R	18/−55/16	N/A	13
Parietal	DIST	PL	L	−21/−64/25	N/A	16
		PreC	R	9/−64/49	N/A (7)	61
		PreC	R	18/−61/28	N/A (31)	24
		iPL	L	−27/−55/40	N/A (7)	58
		iPL	L	−39/−37/43	40	13
		PCG	L	−42/−22/52	3 (40/2)	18
		N/A	L	−15/−76/58	N/A (7)	13
	DIR	PreC	L	−12/73/55	N/A (7)	19
		sPL	L	−18/−58/64	7	10
Occipital	DIST					
	DIR	mOL	R	42/−79/34	N/A (39/19)	20
		iPL	L	−36/−49/46	40 (7)	47
Cerebellum	DIST	CPL	R	24/−64/−23	N/A	46
	DIR	CPL	R	21/−64/−47	N/A	40
		CAL	L	−15/−43/−14	N/A (36/37/35/20)	147
		CAL	R	6/−58/−29	N/A	27

*Broadman Area (BA) of peak voxels are also given (adjacent BA on which some clusters spill over in parenthesis), when applicable, as well as cluster size*.

The main contrast DIST >0 showed activity bilaterally in the temporal cortex, with clusters in the left temporal lobe (*xyz* = −45/−40/1, *Z* = 5.14) and in the right inferior temporal lobe (*xyz* = 45/−58/−14; *Z* = 4.41); in the left frontal cortex, with clusters in the frontal inferior opercule (*xyz* = −33/8/28, *Z* = 4.13), the inferior frontal gyrus (*xyz* = −51/5/34, *Z* = 4.00), and the superior frontal lobe (*xyz* = −24/−4/46, *Z* = 4.91); bilaterally in the parietal cortex, with clusters peaking in the left inferior parietal lobe (*xyz* = −21/−64/25, *Z* = 4.67, *xyz* = −27/−55/40, *Z* = 4.74, and *xyz* = −39/−37/43, *Z* = 4.25) and extending into the precuneus, the inferior parietal lobule, and the superior parietal lobule, a cluster in the left postcentral gyrus (*xyz* = −42/−22/52, *Z* = 4.10), a cluster peaking in the left superior parietal lobe and extending into the precuneus (*xyz* = −15/−76/58, *Z* = 4.07), and a cluster in the right precuneus (*xyz* = 9/−64/49, *Z* = 4.27 and* xyz* = 18/−61/28, *Z* = 4.26); and finally in the right cerebellum, with a cluster in the posterior lobe (*xyz* = 24/−64/−23, *Z* = 4.49).

The main contrast DIR > 0 showed activation in the right temporal cortex, with a cluster peaking in the fusiform gyrus and extending into the posterior parahippocampal gyrus (*xyz* = 33/−37/−17, *Z* = 5.18) and another in the calcarine sulcus (*xyz* = 18/−55/16, *Z* = 4.28); in the left frontal cortex, with a cluster peaking in the precentral gyrus (PCG) and extending into the frontal eye field and the middle frontal gyrus (MFG; *xyz* = −33/−10/58, *Z* = 6.15); in the left parietal cortex, with clusters in the precuneus (*xyz* = −12/−73/55, *Z* = 4.43), the superior parietal lobe (*xyz* = −18/−58/64, *Z* = 3.84), and a cluster peaking in the inferior parietal lobe and extending into the retrosplenial cortex (*xyz* = −36/−49/46, *Z* = 5.12); in the right occipital cortex, with a cluster in the middle occipital lobe (*xyz* = 42/−79/34, *Z* = 4.09); and finally bilaterally in the cerebellum, with a cluster peaking in the left anterior lobe and extending into the posterior parahippocampal gyrus (*xyz* = −15/−43/−14, *Z* = 6.05), another in the right anterior lobe (*xyz* = 6/−58/−29, *Z* = 4.24), and yet another in the right posterior lobe (*xyz* = 21/−64/−47, *Z* = 4.86).

## Discussion

With this study, our goal was to assess the neuroanatomic basis of mental comparisons of distances and directions read off the participants’ cognitive map, by submitting them to the CMRT. As this task is supposed to tap into the same resources as real navigation, we expected hippocampal, parahippocampal, and parietal activations, with a bias in favor of the right side, reflecting the activation of structures usually involved in navigation.

The hippocampus showed no activity. Even though it may seem contradictory and unexpected for a spatial task, it is consistent with many recent studies. For instance, Schinazi et al. (2013) showed that hippocampal size only predicted subjects’ performances in the learning phase, that is, not during navigation in an *already well-known* environment. This could mean that as soon as the environment is known, the hippocampus is not needed anymore to retrieve and process spatial knowledge. This hypothesis is also supported by the results of Spiers and Maguire (2007), who did not find any hippocampal activity in professional London taxi drivers performing a navigation task in a virtual reality (VR) setting of London.

This idea that the role of the hippocampus is mainly to contribute to the construction of a cognitive map (but less so, or even not at all for storage, recovery, and processing) is also shown in Rosenbaum et al. (2004), Shrager et al. (2007), and Shrager et al. (2008). This idea is consistent with recent cognitive models of memory (for a comprehensive view on current knowledge, see Axmacher and Rasch, 2017) and with neurobiological evidence (for a recent review, see Dudai et al., 2015). Because our task tapped into already existing knowledge (the subjects were asked about landmarks of a city where they had lived for at least 2 years), it might have needed little (in this case, infra-threshold) to no hippocampal processing at all.

In addition, the absence of visible significant activity does not mean that nothing happens at all in the hippocampus, but could instead result from a Type II error (failing to detect an actual effect). As reported by Lieberman and Cunningham (2009), wanting at all costs to avoid Type I errors (i.e., false positives) in fMRI research, and especially in tasks allowing multiple cognitive solutions—like ours—may lead to a disproportionate increase in the number of Type II errors.

Lieberman and Cunningham (2009) suggest different types of corrections, in order to avoid this; among them are *p*-value adjustment, cluster size thresholding, and family-wise error corrections. We settled for an intermediate solution, that is, an uncorrected *p-value*, but with cluster-size thresholding set at 8, which, according to Lieberman and Cunningham (2009), is a conservative solution that nonetheless already reduces Type II errors.

Interestingly, the posterior parahippocampal cortex showed significant activity in both hemispheres, but only for direction comparisons.

These results are in line with the double dissociation between the anterior and the posterior hippocampus (linked specifically to the learning and the reading phase, respectively) observed by Iaria et al. (2007) and Schinazi et al. (2013). As our task taps into already known and consolidated memories, it should only activate the most posterior regions of the hippocampal area.

However, our results show a parahippocampal cortex activation and not a posterior activation of the hippocampus. Interestingly, Libby et al. (2012), who analyzed functional connectivity on resting-state fMRI data, found that the connectivity pattern of the hippocampus with the surrounding subregions differs between its anterior and posterior part. More precisely, the anterior hippocampus shows preferential connectivity with the perirhinal cortex, whereas the posterior hippocampus connects preferentially to the parahippocampal cortex (which is more posterior than the perirhinal cortex). This pattern could in turn support the aforementioned cognitive double dissociation observed in the hippocampus by Iaria et al. (2007) and Schinazi et al. (2013), by extending this dissociation to regions downstream of the hippocampus (i.e., perirhinal and entorhinal cortex vs. parahippocampal cortex). In other words, the learning phase could be preferentially supported by anterior regions (anterior hippocampus, perirhinal, and entorhinal cortex), whereas the reading phase would be supported by more posterior regions (posterior hippocampus and parahippocampal cortex).

The reason why distance processing does not also elicit activity in the posterior parahippocampal area remains unaccounted for by the aforementioned hypothesis. Maybe distance processing does not rely enough on allocentric representations, and/or on episodic memory, to elicit suprathreshold parahippocampal activity.

Interestingly, the present body of data is echoed by Epstein (2008) in his review and by Cona and Scarpazza (2019) in their thorough fMRI meta-analysis: instead of being processed primarily in the hippocampus, navigation and spatial long-term memory tasks could preferentially and selectively be processed by the right parahippocampal gyrus and bilateral retrosplenial areas. Although our data show only very little activity in retrosplenial areas and rather bilateral activity in the parahippocampal gyrus, our study confirms an involvement of the parahippocampal gyrus in the cognitive processing required to perform items of the CMRT, which includes most probably spatial long-term memory retrieval and navigational skills (Descloux and Maurer, 2018).

Our data also indicate that parietal regions displayed activity in both hemispheres, most notably in the left superior parietal lobule and bilaterally in the precuneus. In addition, the left parietal cortex was twice as active compared to the right. We also found large displays of activity in the left frontal lobe, more specifically in the MFG and PCG, partially overlapping the frontal eye field. Taken together, these results are compatible with the nonaggregate network model suggested by Ekstrom et al. (2014), which suggested a distributed processing of navigational representations involving the very same brain regions as those shown active in our results.

Given the nature of our task, and of cognition in general (after all, all cognitive processes are embodied, and as such done from a certain point of view), isolating a purely egocentric or allocentric task, or cognitive process, is most probably impossible. Because our task requires subjects to adopt a certain perspective, especially for direction evaluation, and imagining a self-rotation in regard of *external* landmarks, it is both an egocentric and allocentric task. This dual nature, arguably inherent to navigation, is also described by Wilber et al. (2014). These authors describe a vector-based navigation model in which the direction of an unseen navigational goal is computed by combining the local egocentric landmark bearings (i.e., the location of landmarks from the subject perspective) with an allocentric head direction representation. It is relevant to our task, because the two aforementioned subcomponents are also found in the CMRT (respectively, when we asked our subjects to imagine themselves in a given surrounding, and when we asked them to represent themselves and compare three allocentric head directions). Their results, recorded *via* internal electroencephalogram (iEEG), show the posterior parietal cortex (PPC) of rats is highly involved in both these subcomponents, as is the PPC of our human participants while performing our task.

Alexander et al.’s (2020) results also highlight the importance of egocentric modulation in navigational processes. These authors recorded *via* iEEG the activity of cells in rats’ retrosplenial cortex while the animal was freely exploring its 2D environment. They report a large percentage of these cells that have spatial receptive fields responsive to surrounding boundaries with specific orientation and distance, which is coded in an egocentric code. Furthermore, they found a subpopulation of these cells whose activity is synchronize with hippocampal theta waives. The role of theta in memory processes and navigation has been thoroughly studied (for interesting reviews, see Colgin, 2016; Korotkova et al., 2018; Buskila et al., 2019) and will be described within the scope of its various correlates with gamma waves and the CMRT in one of the author’s upcoming study.

Recently, Bicanski and Burgess (2018) developed a simulation modeling how signals of specific neurons types (PC, HDC, and GC among others) can map onto navigation-related high-level cognitive functions. For instance, this model highlighted how GCs could account for the ability of mentally visualizing viewpoints during route planning and taking shortcuts by directly modulating PC activity.

Regarding now the frontal activity, we found a large cluster in the left PCG and in the left MFG. Cona and Scarpazza (2019) found in their meta-analysis that only the left PCG is involved in spatial tasks, whereas its right counterpart is not. More precisely, the left PCG was shown to be involved only in tasks requiring spatial attention and spatial working memory, whereas the MFG was active bilaterally and also involved in mental rotation tasks. Interestingly, we found here the same lateralization pattern for the PCG, but not for the MFG. This could mean that our task relies at least on spatial attention, spatial memory, and spatial rotation. More generally, our results confirm that the left PCG and MFG are involved in mental navigation and spatial processing.

Interestingly, structures found active in the frontoparietal (FP) region in our study suggest that our task also heavily relies on brain structures involved in spatial attention and working memory (WM; Cona and Scarpazza, 2019). In line with their results, we also found a FP pattern of activation, looking very much like the one they observe in their study, that is, an activation pattern resembling the dorsal attention network (DAN; Majerus et al., 2018). This FP circuitry is composed of the frontal eye field, superior parietal lobule, and intraparietal sulcus, and it is usually recruited for perceptive visuospatial tasks, but also for spatial operations made on objects mentally visualized. More generally, the DAN is associated with the internal maintenance of task-related representations (for a review, see Majerus et al., 2018), which obviously means spatial representations for spatial tasks. Given their results, and as part of their conclusions, Cona and Scarpazza (2019) suggest that the DAN is likely to play a key role for working memory, episodic retrieval, and mental imagery.

This is, intuitively, not so striking, when considering how our distance condition requires subjects to recall three known locations (one serving as reference, the other two being targets), and their spatial relationship to each other, and then to keep them in WM to compute the two reference-to-target distances, and then to keep these two distance representations active in order to tell which one is the largest.

The cognitive processes needed to compare two directions, at least given how our task was built, require to keep even more representations active in WM before being able to answer: the subjects had to recall four known locations (two giving a reference direction, the other two being targets) and their spatial relationships to each other.

That the processing of directions is more WM-intensive than the processing of distances is supported by our findings. We indeed found that frontal regions were more than twice as active for condition direction compared to condition distance.

However, there are two important differences to be noted between, on the one hand, our results, and on the other hand, the results of Cona and Scarpazza (2019) and the most common hypothesis (see below) about the lateralization of spatial processing and WM.

The first difference is that we found a significant lateralization of the FP activity, whereas Cona and Scarpazza (2019) found *bilateral* involvement of this FP circuitry. The second difference is that the lateralization we found in the FP area is in favor of the left hemisphere. Consequently, our results do not support a right FP dominance for visuospatial working memory, but indicate a left dominance. Third, we also found in our results some cerebellum activity, which was unexpected. Yakusheva et al. (2007) and Angelaki et al. (2010) studied the vermis region of the cerebellum in macaques. They showed that the vermis is involved in the processing and transformation of self-motion information (stemming from vestibular afferents and vestibular nuclei neurons) and suggested that this region participates in spatial orientation.

Long-term depression (LTD) is a plasticity mechanism that has been hypothesized to be at work in the cerebellum as an error-based learning process (Albus, 1971). L7-PKCI transgenic mice are especially useful in the study of LTD mechanisms, because these animals display a selectively disrupted plasticity of their cerebellar Purkinje cells synapses, thus resulting in impaired LTD. Burguière et al. (2005) have shown that L7-PKCI transgenic mice did not perform as well as their wild-type peers in the Morris water maze and Starmaze tasks (which required them to find an escape platform while swimming in opaque water). The authors thus suggest that cerebellar LTD is involved is sensorimotor optimization and spatial navigation.

Rochefort et al. (2011) also worked with wild-type mice, and with strains of transgenic L7-PKCI animals. Their hippocampal CA1 cells (place cells) activity was recorded as they were exploring a circular arena, then their behavior was recorded during a path integration task (in total darkness) in a Morris water maze. Following cue manipulation in the first task, the authors showed that the firing fields of hippocampal place cells were not efficiently controlled by self-motion cues in the transgenic mice; in the water maze task, which required the use of self-motion cues, those mice were also impaired compared to the wild-type mice. Interestingly, Rochefort et al. (2011) also found that L7-PKCI mice had a significantly lower proportion of place cells than their wild-type peers. These findings suggest that plasticity-dependent mechanisms are involved in the processing of self-motion information in mice and, maybe more importantly, that these mechanisms have a direct impact on hippocampal cell recruitment and spatial representations.

Together, all these studies describing cerebellar involvement in spatial orientation in animals suggest an important and seemingly underestimated role of the cerebellum in human spatial cognition. This is probably related to its function: the cerebellum seems involved in the transformation of self-motion cues into rotational information that could be of use to build higher-level representations of space. In order to investigate the role of the cerebellum in the processing of such cues in humans, it would of course be necessary to allow subjects to move freely while their cerebellar activity is being recorded. To the authors’ knowledge, such telemetry techniques are either nonexisting, not precise enough, or not applicable on the large environmental scales that are relevant to study human spatial navigation.

Regarding occipital activations, they were the smallest in terms of cluster size and number of active voxels. Interestingly, though, this lobe displayed activation only for distance processing. In general, a top-down process explains occipital activation in mental imagery tasks (O’Craven and Kanwisher, 2000). This this top-down activation theory does not explain why distance processing, and not direction processing, seemed to rely on the occipital lobe. This is even truer when considering how direction processing relies probably even more on mental imagery than distance comparisons.

Finally, the neat right-sided lateralization we expected was not observed. On the contrary, every brain region showed more left than right activation, with an exception for the occipital lobe and the cerebellum, which both showed more right- than left-sided activation. This would tend to show that there is no clear right-sided specialization for navigation and that, depending on the nature of the task, the left hemisphere may even be more involved than the right (Lambrey et al., 2003).

## Conclusion

In this study, our aim was to assess which brain structures were involved in reading and processing the information stored in the cognitive map of a well-known environment. To that effect, we submitted our participants to a mental navigation task, the CMRT; we assumed that this mental map-processing task would tap into the same brain structures as real navigation. The main exploratory focus here was to identify if partly different brain structures process distances and directions. Generally, we have shown that our task indeed involves brain areas usually associated with navigation, that is, a circuitry involving the posterior parahippocampal cortex, the parietal lobule, the precuneus, and the PCG and MFG. We also found areas that are far less often associated with spatial navigation in humans, in particular the cerebellum and the occipital cortex. In contrast, hippocampal activity was low, and this is in line with the fact that the task required the recall of old, well-established spatial relations. As for the processing of distances vs. directions, only direction comparisons elicited significant bilateral activity in the posterior parahippocampal cortex. It is not clear why distance comparisons did not cause a similar pattern of activity; this will require further investigations, especially through methods with better temporal resolution, allowing the analysis of dynamic connectivity patterns involved in the processing of distance and direction. It remains true that, as pointed out by Epstein et al. (2017, p. 1509), “an important question for future research is how distance and direction are processed in highly familiar environments, where the hippocampus is not as needed for navigation.”

## Data Availability Statement

The raw data supporting the conclusions of this article will be made available by the authors, without undue reservation.

## Ethics Statement

The studies involving human participants were reviewed and approved by FPSE Faculty Ethical Committee (Commission Facultaire D’éthique De La FPSE). The patients/participants provided their written informed consent to participate in this study.

## Author Contributions

IF: conceptualization, methodology, investigation, data curation, writing—original draft, visualization, and formal analysis. VD: methodology. AS: formal analysis. RM: supervision, writing—review and editing, project administration, and funding acquisition.

## Conflict of Interest

Since the 1st of February 2020, the co-author IF has been employed by Frontiers Media SA. IF declared his affiliation with Frontiers, and the handling Editor states that the process nevertheless met the standards of a fair and objective review.

The remaining authors declare that the research was conducted in the absence of any commercial or financial relationships that could be construed as a potential conflict of interest.

## Footnotes

^1^www.fil.ion.ucl.ac.uk/spm
